# Methotrexate in oligoarticular persistent juvenile idiopathic arthritis

**DOI:** 10.1186/1546-0096-12-S1-P139

**Published:** 2014-09-17

**Authors:** Gerd Horneff, Ivan Foeldvari, Gerd Ganser, Angelika Thon, Heinrike Schmeling

**Affiliations:** 1 Asklepios Clinic, Sankt Augustin, Germany; 2 Hamburg Centre for Pediatric and Adolescence Rheumatology, Hamburg, Germany; 3 North West German Centre of Rheumatology and Orthopaedics, Sendenhorst, Germany; 4 Medizinische Hochschule Hannover, Hannover, Germany; 5 Department of Paediatrics, Alberta Children’s Hospital, Calgary, Canada

## Introduction

Methotrexate (MTX) is recommended for children with juvenile idiopathic arthritis (JIA) with persistent oligoarticular course with high disease activity and features of poor prognosis or if they failed intraarticular glucocorticoid steroid injections following the 2011 ACR recommendations for the treatment of JIA. To date efficacy and safety of MTX for this JIA category have not been well studied.

## Objectives

Evaluation of efficacy and safety of MTX in children with oligoarticular persistent JIA.

## Methods

Baseline demographics, clinical characteristics and disease activity parameters have been prospectively documented in the German JIA BIKER Register. Efficacy was determined using the PedACR response criteria and the JADAS-10 until the last documentation or start of any biologic agent. Safety assessments were based on adverse events reports from the responsible physician.

## Results

343 patients with a total of 2039 visits were identified (894.7 total patient-years). 67% (n=231) were females. The median age at JIA onset was 4.7 (quartile (Q) 25/75 2.5/9.2). At treatment initiation the median age was 8.0 years (Q25/75 4.3/12.0) and the disease duration 1.1 years (Q25/75 0.5/3.1). ANA positivity has been found in 63.0%, HLB27 positivity in 11.1%. Prior MTX therapy 90.1% of children have been treated with nonsteroidal anti-inflammatory drugs (NSAIDs), 46.1% have received intraarticular glucocorticoid steroid injections. 88.3 of children were treated concomitantly with NSAIDs, 11.5% with corticosteroids and 2.6% with other disease-modifying antirheumatic drugs.

A high proportion of patients showed a significant response to treatment. Compared to baseline disease activity parameters at MTX initiation, at month 6 70.5%/61.0%/41.5%, at month 12 73.9%/63.9%/43.5% and at month 24 83.6%/80.0%/69.7% of patients met the PedACR response criteria of 30%/50%/70%. The median JADAS-10 at treatment start was 10.0 and decreased markedly to 2.1 at month 6, 1.4 at month 12 and 1.0 at month 24. At month 6 and month 12, 83 patients (33%) and 96 patients (42%) reached preliminary criteria for inactive disease.

113 patients experienced 216 adverse events (AE,24.7/100 patient-years). Four were reported as serious (0.4/100 patient-years (py)). The most common reported AEs were nausea/vomiting (8.5/100 py), abnormal liver enzymes (4.7/100 pj) and mild to moderate infections (3.6/100 pj). 48 children (14%) suffered from uveitis prior treatment. None of these patients flared with uveitis. Four new uveitis index cases (1.1%) were reported while on treatment.

37 patients started a biologic agent, 16 of them switched from MTX to biologics while 21 patients were treated in combination with MTX. Treatment has been discontinued in 134 children for the following reasons: Inefficacy 3.2%, adverse events 9.8%, remission 24.3%, patients wish 16.5%, others 2.9%. 17 of 84 children (20.2%) who stopped MTX because of remission flared and re-started MTX.

## Conclusion

Methotrexate appears to be highly effective in oligoarticular persistent JIA. The treatment is safe and well-tolerated. Few patients discontinued due to intolerance or inefficacy.

## Disclosure of interest

None declared

